# ARID1A Is a Prognostic Biomarker and Associated with Immune Infiltrates in Hepatocellular Carcinoma

**DOI:** 10.1155/2022/3163955

**Published:** 2022-01-04

**Authors:** Yuanyuan Feng, Xinfang Tang, Changcheng Li, Ying Su, Xiaoyu Wang, Ning Li, Anna Zhang, Feng Jiang, Chuyan Wu

**Affiliations:** ^1^Department of Oncology, The Affiliated Lianyungang Oriental Hospital of Kangda College of Nanjing Medical University, The Affiliated Lianyungang Oriental Hospital of Xuzhou Medical University, The Affiliated Lianyungang Oriental Hospital of Bengbu Medical College, Lianyungang 222042, China; ^2^Department of Nephrology, The Affiliated Lianyungang Oriental Hospital of Kangda College of Nanjing Medical University, The Affiliated Lianyungang Oriental Hospital of Xuzhou Medical University, The Affiliated Lianyungang Oriental Hospital of Bengbu Medical College, Lianyungang 222042, China; ^3^Department of Radiology, The Affiliated Lianyungang Oriental Hospital of Kangda College of Nanjing Medical University, The Affiliated Lianyungang Oriental Hospital of Xuzhou Medical University, The Affiliated Lianyungang Oriental Hospital of Bengbu Medical College, Lianyungang 222042, China; ^4^Department of Personnel Division, The Affiliated Lianyungang Oriental Hospital of Kangda College of Nanjing Medical University, The Affiliated Lianyungang Oriental Hospital of Xuzhou Medical University, The Affiliated Lianyungang Oriental Hospital of Bengbu Medical College, Lianyungang 222042, China; ^5^Department of Gynaecology and Obstetrics, The Affiliated Lianyungang Oriental Hospital of Kangda College of Nanjing Medical University, The Affiliated Lianyungang Oriental Hospital of Xuzhou Medical University, The Affiliated Lianyungang Oriental Hospital of Bengbu Medical College, Lianyungang 222042, China; ^6^Department of Neonatology, Obstetrics and Gynecology Hospital of Fudan University, Shanghai 200011, China; ^7^Department of Rehabilitation Medicine, The First Affiliated Hospital of Nanjing Medical University, Nanjing 210000, China

## Abstract

**Objective:**

ARID1A has been discovered as a potential cancer biomarker. But its role in hepatocellular carcinoma (HCC) is subject to considerable dispute.

**Methods:**

The relationship between ARID1A and clinical factors was investigated. Clinicopathological variables related to overall survival in HCC subjects were identified using Cox and Kaplan–Meier studies. The connection between immune infiltrating cells and ARID1A expression was investigated using the tumor Genome Atlas (TCGA) dataset for gene set enrichment analysis (GSEA). Finally, a cell experiment was used to confirm it.

**Results:**

The gender and cancer topography (T) categorization of HCC were linked to increased ARID1A expression. Participants with advanced levels of ARID1A expression had a worse prognosis than someone with lower levels. ARID1A was shown to be a risk indicator of overall survival on its own. ARID1A expression is inversely proportional to immune cell infiltration. In vitro, decreasing ARID1A expression substantially slowed the cell cycle and decreased HCC cell proliferation, migration, and invasion.

**Conclusion:**

The expression of ARID1A could be used to predict the outcome of HCC. It is closely related to tumor immune cell infiltration.

## 1. Introduction 

The global incidence rate of hepatocellular carcinoma (HCC) is extremely high, with an annual increase [1]. Over the course of five years, the overall survival rate is less than 5% [2]. There are currently just a few early detection techniques for HCC [3]. In earlier studies, numerous mutated genes in hepatocellular cancer have been identified, with ARID1A being the most contentious [4]. ARID1A has been found to affect metastasis and tumor cell proliferation in functional investigations [5]. In gynecological tumor cells lacking ARID1A, cell growth and colony formation increased [6]. In fact, suppressing ARID1A increased the invasion and migration of liver cancer cells. However, additional results suggest that ARID1A has a more complicated role in carcinogenesis and that some of the SWI/SNF components are carcinogenic in certain instances [7, 8]. All of these results point to ARID1A participating in tumor start, particularly in the development of liver tumor.

Then, using Cancer Genome Atlas (TCGA) database, this study sought to determine the prognostic and diagnostic validity of ARID1A expression level in HCC. The biological route of the ARID1A regulatory network linked with hepatocarcinogenesis was investigated further using gene set enrichment analysis (GSEA). Then, using hepatoma cell lines, we investigated the connection between ARID1A expression and immune infiltration.

## 2. Materials and Methods

### 2.1. Data and Analysis

To evaluate the degree of ARID1A expression in hepatic tumor and normal hepatic samples, the TCGA database was used. A total of 374 instances of tumor and 50 instances of adjacent liver tissue with ARID1A mRNA expression relevant data were downloaded from the TCGA database (http://cancergenome.nih.gov/). R3.6.1 software was used to examine the difference in ARID1A mRNA expression performance between the two groups.

### 2.2. Gene Set Enrichment Analysis

GSEA is a technique for determining if a collection of preset genes exhibits statistical differences in expression between upper and lower groups [9, 10]. GSEA software has been used to generate datasets and label phenotypic files. The phenotypic designations ARID1A-low and ARID1A-high are used. For each study, 1000 permutations of gene sets were conducted. The gene determines the standard *P* value.

### 2.3. Cell Culture

Human HCC cell lines Huh7, HepG2, Alex, Bel-7402, Hep3B, 97H, LM3, and human normal liver cell line Hl-7702 were purchased from the Institute of Cell Research, Chinese Academy of Sciences, and they were grown in Dulbecco's modified Eagle's medium (Gibco, Grand Island, NY, USA) supplemented with 10% fetal bovine serum (FBS, Gibco, USA) and 1% penicillin-streptomycin in a 37°C incubator containing 5% CO_2_.

### 2.4. Cell Counting Kit-8 (CCK-8) Experiment

The vitality of the cells was determined using the Cell Counting Kit-8 (CCK-8) as directed by the manufacturer (Beyotime Institute of Biotechnology, China). The specified quantities of cells were cultured onto 96-well plates and grown for 5 days with the culture media replaced every 2 days (counted using a Cellometer Mini, Nexcelom Bioscience, Massachusetts, USA). Each well received an aliquot of 100 *µ*l of Cell Counting Kit-8 solution, which was dripped and incubated for 1 hour before the absorbance value was measured at 450 nm (Elx800; BioTek Instruments, Inc., Winooski, VT, USA).

### 2.5. Colony Formation Assay

A total of 500 97H cells or LM3 cells were seeded in 6-well plates for plate colony formation. The cells were fixed with 4% formaldehyde and stained with 1% crystal violet (Sigma Aldrich, USA) before being imaged 14 days later. The colonies were counted and analyzed using the Alpha Innotech imaging equipment (Singapore Alphatron Asia Co., Ltd.).

### 2.6. Immune Landscape Estimation and Correlation Analysis

CIBERSORT (https://cibersortx.stanford.edu/) was used to infer the 22 immune cell values in TCGA cohort by evaluating the percentage of cases with the expression of Leukocyte signature matrix (LM22) classic genes using the R package “corrplot” with 1000 permutations [11]. For the following analysis, cases having a CIBERSORT result of *P* < 0.05 were chosen. To illustrate the variations in immune cell invasion between the two groups, violin plots were created in R using the “vioplot” package. Spearman's correlation study in R language was used to investigate the relationship between the discovered gene indicator and the numbers of invading immune cells. The resultant correlations were displayed using the “ggplot2” package's chart method.

### 2.7. Immunity Analysis and Gene Expression

CIBERSORT [11, 12], ESTIMATE [13], MCPcounter [14], single-sample gene set enrichment analysis (ssGSEA) [15], and TIMER algorithms [16] were evaluated to measure cellular function or immune effects between low- and high-risk groups according to ARID1A expression. A heatmap was used to reveal variations in immune reaction under various algorithms. Furthermore, ssGSEA was utilized to compare and identify the tumor-infiltrating immune cell subsets. Previous literature also yielded a possible immunological checkpoint.

### 2.8. The Human Protein Atlas (HPA) Website

This database (http://www.proteinatlas.org/) includes tissue and cell expression levels of approximately 20,000 human proteins, as well as information on protein distribution in various human tissues and organs [17, 18]. This website was utilized to look at the protein of ARID1A expression in cancerous and normal liver tissues.

### 2.9. RNA Interference

ARID1A was knocked out via RNA interference. Each well of a six-well plate was filled with liver cancer cells. After 24 hours, the cells in each well were transfected for 48 hours using 3.75 *µ*l lipofectamine 3000 and 5 *µ*l siRNA oligo (20 *μ*M). Cells were digested again and submitted to the appropriate tests (ARID1A siRNA sequence: siRNA#1 GGAGCUAUCUCAAGAUUCAUU; siRNA#2 AGUUCUAGGUUCAGUUGAAGU).

### 2.10. RT-PCR

ARID1A has a forward primer sequence of ACTCCATGGGGAGCTAGGT and a reverse primer sequence of CACCCATGGTTTATGCCT. The Ct rates of the discovered genes were compared to the Ct values of GAPDH, an internal control gene. Every experiment was carried out a total of three times. The relative expression was calculated using the 2^−ΔΔ*Ct*^ technique.

### 2.11. Statistical Analysis

Regression analysis and Kolmogorov–Smirnov analysis were used to evaluate the relationship between clinical and the expression of ARID1A. The cox regression and Kaplan–Meier methods were used to identify treatment variables linked to complete survival in patients with hepatocellular carcinoma. The role of ARID1A expression in survival was next investigated using multivariate Cox analysis in conjunction with clinical characteristics. The median results were used to determine if ARID1A expression was low or high. The average data point of expression level was chosen as dividing point, and all of these cases were divided into two groups based on their ARID1A expression levels: high or low. For every statistical analysis, R software (v3.6.1) was utilized.

## 3. Results

### 3.1. ARID1A Expression in HCC

**Figure 1:**
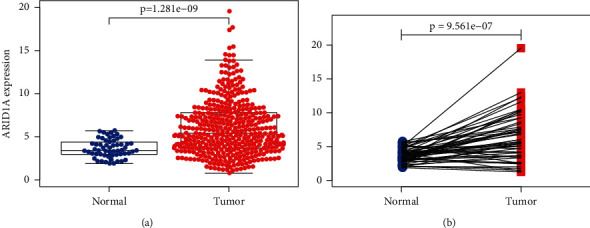
ARID1A expression in liver cancer tissues and normal tissues. (a) Scatter plot and (b) paired difference plot.

The ARID1A mRNA expression degree between normal tissues and tumor tissues was investigated using TCGA data and a bioinformatics method. Scatter plots and paired difference plots were utilized. ARID1A mRNA levels were significantly greater in liver tumor tissues than in normal tissues, as illustrated in Figures 1(a) and 1(b) (*P* < 0.05).

### 3.2. Clinical Characteristics and ARID1A Expression

**Figure 2:**
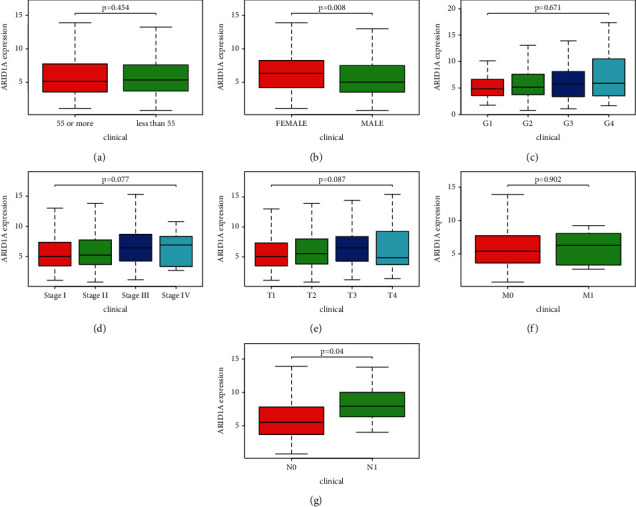
Association of ARID1A expression with clinical variables. (a) Age, (b) gender, (c) grade, (d) stage, (e) tumor topography, (f) distant metastases, and (g) lymph node metastasis.

Clinical information from 377 HCC patients in the TCGA was examined, including the patient's age, gender, histological grade, stage, tumor topography, distant metastasis, lymph node (TMN) classification, survival time, and survival status. ARID1A expression was significantly related to gender (*P*=0.008) and lymph node categorization (*P*=0.04), as shown in Figures 2(a)–2(g). ARID1A expression was related to gender (OR = 0.35, female versus male) and T classification (OR = 1.92, T3 versus T1) in liver cancer (Table 1).

### 3.3. Multivariate Analysis and Survival Results

**Figure 3:**
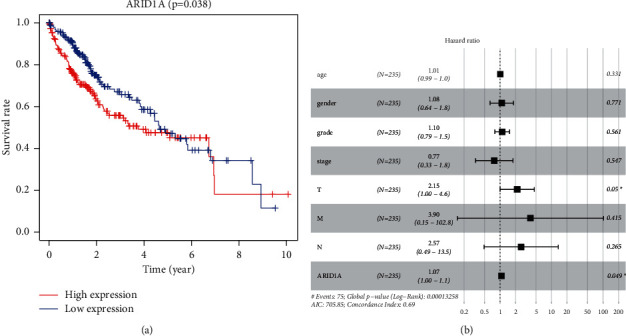
(a) ARID1A expression and overall survival in liver cancer patients in TCGA cohort. (b) MultiCox analysis regression analysis identifying prognostic variables with HR with 95% CI and *P* values.

As shown in Figure 3, high expression level was associated with disappointing overall survival (*P*=0.038) (Figure 3). The univariate analysis revealed that overexpression of ARID1A was strongly related to negative clinical outcome [hazard ratio (HR): 1.0985; 95 percent confidence interval (CI): 1.0341–1.1669; *P*=0.0023, Table 2]. Clinical variables linked to poor survival were included in the stage, T, and M classifications (Table 2). The multivariate analysis took into account all of the variables. Excellent ARID1A expression was shown to be a self-regulating risk key to overall survival (HR = 1.0682, 95 percent CI: 1.0004–1.1406, *P*=0.0487) and T categorization (HR = 2.1529, 95 percent CI: *P*=0.0499, 1.0004–4.6332).

### 3.4. ARID1A-Related Signaling Pathways Are Identified Using GSEA

**Figure 4:**
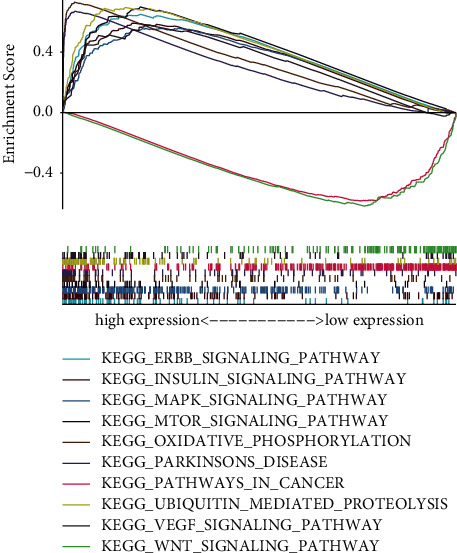
Enrichment plots from gene set enrichment analysis (GSEA).

GSEA searches for physiological processes that are activated differentially in hepatocellular carcinoma by identifying ARID1A-associated signal pathways. The decrease and upwards ARID1A expression datasets were compared in GSEA. The nominal *P* value of 0.05 and the FDR *q* value of 0.25 are determined to be substantially enriched. GSEA discovered significant variations in the enrichment of the MSigDB dataset (h.all.v6.2.symbols.gmt). The most significantly enriched signaling pathways were determined based on their standardized enrichment markers. As shown in Figure 4, cancer signaling, ERBB signaling, mTOR signaling, insulin signaling, VEGF signaling, MAPK signaling, ubiquitin signaling, and Wnt signaling were all associated with the high expression phenotype of ARID1A, while the low expression of ARID1A was associated with Parkinson's disease and oxidative signaling.

### 3.5. Association between the ARID1A Expression and Immune Cells That Infiltrate Tumors

**Figure 5:**
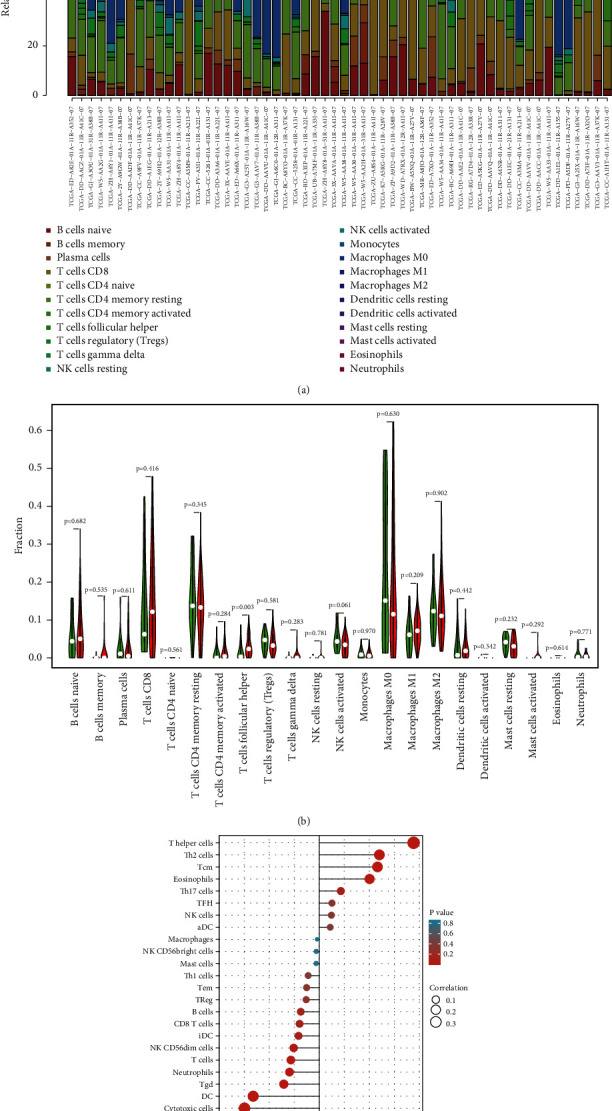
(a) Summary of estimated compositions of 22 immune cell subtypes from the CIBERSORT algorithm in HCC patients. (b) Comparison of 22 immune cell subtypes between low- and high-risk samples. (c) The correlation between ARID1A and infiltrating immune cells in patients with HCC.

The relative number of 22 types of immune cells for every sample was estimated using the CIBERSORT method and compared between the two groups. In the HCC samples, the frequency proportions of 22 different kinds of immune cells were determined (Figure 5(a)). The percentage of T cells follicular helper was substantially greater in the high-risk group (*P*=0.003) than in the low-risk group (Figure 5(b)). Spearman's correlation was used to assess the relationship between immune cells invading LHIC and ARID1A expression. As shown in Figure 5(c), ARID1A was positively associated with activated eosinophils (*r* = 0.201, *P* < 0.001), T helper cells (*r* = 0.377, *P* < 0.001), Tcm (*r* = 0.233, *P* < 0.001), and Th2 cells (*r* = 0.241, *P* < 0.001) and negatively correlated with cytotoxic cells (*r* = −0.299, *P* < 0.001), DC (*r* = −0.264, *P* < 0.001), neutrophils cells (*r* = −0.119, *P*=0.022), NK CD56dim cells (*r* = −0.102, *P*=0.049), pDC (*r* = −0.346, *P* < 0.001), and Tgd cells (*r* = −0.142, *P*=0.006).

### 3.6. Immune Infiltration and ARID1A Expression

**Figure 6:**
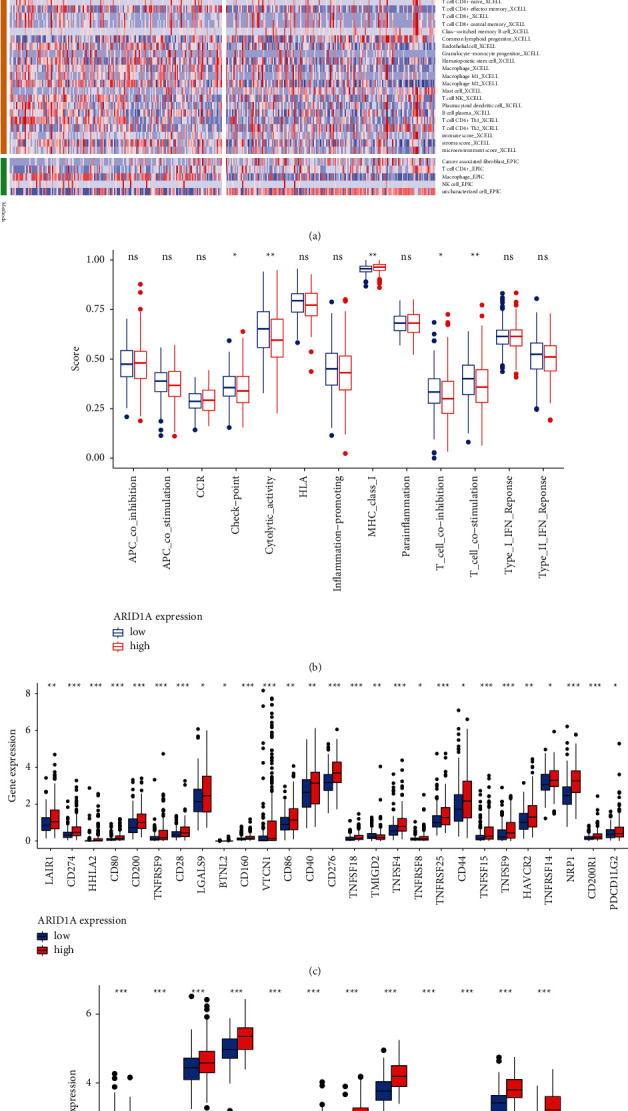
(a) Heatmap for immune responses based on CIBERSORT, ESTIMATE, MCPcounter, ssGSEA, and TIMER algorithms among high and low-risk group. (b) ssGSEA for the association between immune cell subpopulations and related functions. (c) Expression of immune checkpoints among high- and low-risk ARID1A groups. (d) The expression of m6A-related genes between high- and low-risk ARID1A group.

Figure 6(a) shows a heatmap of immunological responses depending on the algorithms. According to ssGSEA of TCGA-HCC data, correlation analysis of immune cell subtypes and associated activities showed that T cell functions such as cytolytic, checkpoint, MHC class-1, coinhibition, and costimulation were substantially different between the two groups (Figure 6(b)). Because checkpoint inhibitor-based immunotherapies are so important, we looked at the differences in immune checkpoint expression between the two groups. Between the two groups of patients, we discovered a significant variation in the expression of PDCD1LG2, CD274, LAG3, and VTCN1, among other genes (Figure 6(c)). There were significant variations in the expression of 12 methyltransferases, such as ZC3H13, METTL14, and HNRNPC, when the m^6^A expression related mRNA was compared between the two groups (Figure 6(d)).

### 3.7. Effects of ARID1A Deficiency on HCC Cell Proliferation, Migration, and Invasion

**Figure 7:**
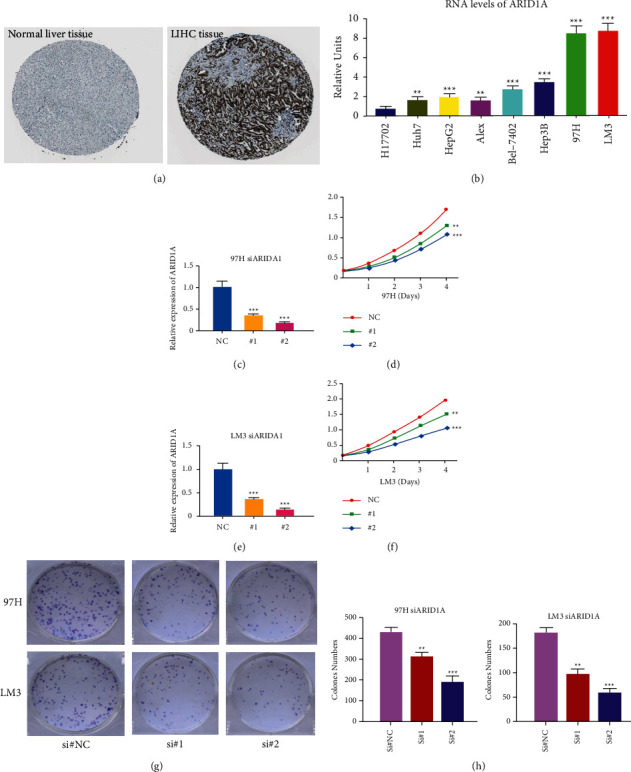
Experimental verification of ARID1A. (a) Immunohistochemical images from the HPA database show ARID1A protein expression in normal live tissues and HCC tissues. (b) Analysis of ARID1A expression in HCC and normal liver cell lines. (c) RT-PCR was used to detect the efficiency of knockdown for ARID1A in 97H. (d) 97H were treated with siRNA against ARID1A for 24 hours and then subjected to the CCK-8 assay. (e) RT-PCR was used to detect the efficiency of knockdown for ARID1A in LM3. (f) LM3 were treated with siRNA against ARID1A for 24 hours and then subjected to the CCK-8 assay. (g–h) 97H and LM3 were treated with siRNA against ARID1A for 24 hours and then subjected to the colony formation assay. ^*∗*^*P* < 0.05, ^∗∗^*P* < 0.01, and ^∗∗∗^*P* < 0.001.

Using the HPA website, we looked at the expression of ARID1A in normal and tumor tissue. The amount of ARID1A protein expression in liver cancerous tissue was considerably higher than in noncancerous tissue, according to our findings (Figure 7(a)). RT-PCR analysis was performed in Hl-7702 human benign HCC line and various liver tumor cell lines to investigate the RNA expression of ARID1A in liver cancer (Huh7, HepG2, Alex, Hep3B, Bel-7402, 97H, and, LM3). ARID1A expression was much greater in HCC than in normal cell lines, as illustrated in Figure 7(b). Compared to Hl-7702 cells as the control, the fold change values of ARID1A RNA expression in HCC cells were 1.723 (Alex, *P* < 0.001), 1.831 (Huh7, *P* < 0.001), 2.078 (HepG2, *P* < 0.001), 3.011 (Bel-7402, *P* < 0.001), 3.516 (Hep3B, *P* < 0.001), 8.208 (97H, *P* < 0.001), and 8.412 (LM3, *P* < 0.001). We developed two siRNAs targeting the CDS or 3′-UTR of ARID1A to suppress its expression to mechanistically validate the results of expression analysis. According to CCK8 staining findings, downregulation of ARID1A RNA expression reduced the capacity of the liver tumor cells LM3 and 97H to proliferate (Figures 7(c)–7(f)). The second siRNA had the greatest effect on ARID1A expression and prevented HCC cells from multiplying. According to the colony formation test, the second more powerful siRNA targeting ARID1A was similarly efficient in suppressing the growth of liver tumor cell lines LM3 and 97H (Figure 7(g)).

## 4. Discussion

ARID1A is overexpressed in HCC, according to the current study's examination of TCGA data. ARID1A has previously been demonstrated to play an important function as a negative regulator, and its expression degree was linked to outcome in tumor patients with gastric, prostate, and lung cancers [19, 20]. Zhao et al. on the other hand, found that 83 percent of tumors overexpress ARID1A when compared to neighboring liver tissues [21]. Between the normal and experimental groups, we compared two types of plots. The distance map was the first chart, and the matching chart was the second, both of which showed a significant difference between the two groups. From a bioinformatics perspective, our results suggest that ARID1A is already highly expressed in hepatocellular carcinoma and may play a role in tumor start.

Then, we looked at its links to clinical and anticipated patient characteristics and found that it was significantly related to gender (*P* < 0.05) and lymph node stage (N) categorization (*P* < 0.05). Male and female liver cancers have a 3–5 : 1 incidence ratio [22], and our findings reveal gender disparities as well. Our research included more male patients than female patients, while female patients had greater ARID1A expression. ARID1A may be lost later in the progression of liver cancer, according to certain research [23–25]. Our findings also indicate that while expression in the N1 stage is greater than in the N0 stage, it suddenly dropped in the T4 stage, becoming even lesser than in the T1 phase. Each of these findings revealed ARID1A may be important in the development of HCC [4].

Patients with elevated ARID1A expression in their hepatocellular carcinoma tissues had a short prognosis. The low and high expression communities crossed the graph in the later stages of the survival chart, which may be caused by ARID1A variation or loss in the advanced stages of liver tumor, indicating that ARID1A could have complex roles in the development and occurrence of HCC. ARID1A failure has previously been related to tumor growth but has little predictive significance in HCC patients, according to previous study [18, 21]. ARID1A was shown to enhance cancer initiation in part due to its transcriptional limitation of Cyp2e1, which resulted in increased reactive oxygen species (ROS) production [4]. ARID1A expression seems to have a significant role in overall survival in MultiCox and UniCox analyses, suggesting that it may be utilized as a therapeutically useful self-governing risk indicator for hepatocellular carcinoma.

GSEA was used with TCGA data to better examine ARID1A's involvement in HCC. Low expression of ARID1A is mostly linked to Parkinson's disease signaling system and oxidative signaling system. The MAPK and VEGF signaling systems are important in the proliferation and metastasis of liver tumor [26]. The PI3K/Akt/mTOR and PKB/Akt signaling systems are important in the genesis, progression, and outcome of liver tumor [27]. By stimulating its downstream genes, the Wnt signaling system influences the incidence and progression of liver tumor [28–30]. Multiple pathways have been demonstrated to have a significant and central function in HCC in many studies. Sorafenib, a tyrosine kinase inhibitor (TKI), has been authorized for the treatment of advanced liver cancer patients. The development of drug resistance, however, limits the effectiveness of sorafenib, and the main neuronal isoform of RAF, BRAF, and MEK pathways plays a crucial and important role in HCC escape from TKI action [31]. As a result, alternate routes and combination therapy play a significant role in the treatment of liver cancer. ARID1A is associated with a variety of HCC related pathways and may be a potential target of drug resistance.

At present, the treatment of liver cancer has entered the era of immunotherapy. A large number of articles have focused on the connection between ARID1A and immune cell microenvironment and immunotherapy. Some studies suggest that ARID1A have an essential function role in the formation of lymphocytes. In animal studies, it is found that the absence of ARID1A in early lymphoid progenitor cells of mice leads to the obvious stagnation of early T cell development [32]. Our findings further indicate that the ARID1A expression is significantly linked to T helper cells. A pan cancer study suggests that changes in ARID1A can be used as a biomarker for immunotherapy outcomes [33]. Our study also suggests that the expression of ARID1A is linked to immune checkpoint. Above all, significant ARID1A expression in tumor cell lines was verified. Furthermore, reducing ARID1A expression substantially slowed the cell cycle and decreased HCC cell proliferation, emigration, and infiltration in vitro.

The mutation impact of ARID1A in tumors has been the subject of many research. Using the TCGA database, this is the first research to show a connection between ARID1A expression and HCC survival and immune infiltration, as well as to indicate that ARID1A could offer inner prognostic components to inhibit HCC clinical characteristics. Despite this, the research had several flaws. The research contains flaws related to retrospective data gathered from the TCGA databases since it is a retrospective study. For external validation, large-scale multicenter prospective cohorts are required. In addition, in vivo studies should be conducted in the future to validate the results.

## 5. Conclusion

According to the study, ARID1A is involved in the aggressiveness and carcinogenesis of HCC and therefore could be played as a prognostic biomarker in individuals with HCC. Because ARID1A expression is related to immune cell invasion, it could be utilized as a screening mean to explore HCC sufferers who would gain from immunotherapy. Some results (Figures 1–4 and Tables 1 and 2) have been uploaded to the website as a preprint (https://www.researchsquare.com/article/rs-146505/v1) [34], and immune infiltration analysis and experimental verification have been added.

## Figures and Tables

**Table 1:** Logistic regression of ARID1A expression and clinical pathological characteristics.

Clinical characteristics	Total	Odds rate in ARDI2 expression	*p* value
Age (<55 versus ≥55)	366	1.13(0.75–1.83)	0.412
Gender (male versus female)	377	0.35(0.24–0.57)	<0.001
Grade (G2 versus G1)	235	1.24(0.73–2.50)	0.331
Grade (G3 versus G1)	179	1.62(0.81–3.27)	0.109
Grade (G4 versus G1)	68	1.59(0.46–1.93)	0.625
Stage (2v1)	262	1.11(0.62–1.61)	0.875
Stage (3v1)	261	1.26(1.03–2.72)	0.053
Stage(4v1)	200	1.79(0.45–6.92)	0.432
T (T2 versus T1)	280	1.18(0.79–2.23)	0.351
T (T3 versus T1)	266	1.92(1.03–3.22)	0.014
T (T4 versus T1)	198	0.81(0.23–2.35)	0.639
M (M1 versus M0)	276	1.35(0.29–6.83)	0.731
N (N1 versus N0)	261	3.16(0.78–20.23)	0.178

**Table 2:** Univariate and multivariate analysis of the relationship between ARID1A expression and overall survival among LIHC patients.

Id	UniCox				MultiCox			
	HR	HR.95L	HR.95H	*P* value	HR	HR.95L	HR.95H	*P* value
Age	1.0050	0.9869	1.0235	0.5912	1.0095	0.9904	1.0290	0.3310
Gender	0.7801	0.4872	1.2492	0.3013	1.0806	0.6417	1.8196	0.7706
Grade	1.0172	0.7459	1.3871	0.9143	1.1027	0.7933	1.5327	0.5608
Stage	1.4172	1.2349	1.6265	≤0.001	0.7728	0.3338	1.7894	0.5474
T	1.8044	1.4341	2.2702	≤0.001	2.1529	1.0004	4.6332	0.0499
M	3.8498	1.2068	12.2813	0.0228	3.8978	0.1478	102.7721	0.4151
N	2.0218	0.4939	8.2761	0.3276	2.5713	0.4881	13.5439	0.2653
ARID1A	1.0985	1.0341	1.1669	0.0023	1.0682	1.0004	1.1406	0.0487

## Data Availability

The datasets used during the present study are available from TCGA (https://portal.gdc.cancer.gov/repository) database.
